# Will the embedded semantic radicals be activated when recognizing Chinese phonograms?

**DOI:** 10.3389/fnhum.2025.1550536

**Published:** 2025-06-13

**Authors:** Meng Jiang, Xueyao Pan, Xia Wang, Qi Luo

**Affiliations:** ^1^College of Language Intelligence (College of General Education), Sichuan International Studies University, Chongqing, China; ^2^Language & Brain Research Center, Sichuan International Studies University, Chongqing, China; ^3^Chongqing Shapingba District International Joint Institute of Brain Computer Language Interface, Chongqing, China; ^4^School of Foreign Languages and Literature, Chongqing Normal University, Chongqing, China; ^5^School of English Studies, Sichuan International Studies University, Chongqing, China

**Keywords:** phonograms recognition, embedded semantic radical, sub-lexical semantic activation, frequency, Chinese characters

## Abstract

**Introduction:**

A majority of Chinese characters are phonograms composed of phonetic and semantic radicals that serve different functions. While radical processing in character recognition has drawn significant interest, there is inconsistency regarding the semantic activation of embedded semantic radicals, and little is known about the duration of such sub-lexical semantic activation.

**Methods:**

Using a priming character decision task and a between-subjects design, this study examined whether semantic radicals embedded in SP phonograms (semantic radicals on the left and phonetic radicals on the right) can be automatically activated and how long such activation persists. We manipulated semantic relatedness between embedded radicals and target characters, prime frequency, and stimulus onset asynchronies (SOAs).

**Results:**

Facilitatory effects were observed on targets preceded by low-frequency primes at an SOA of 500 ms. No significant priming effects were found at SOAs of 100 ms or 1000 ms, regardless of prime frequency.

**Discussion:**

These findings suggest that sub-lexical semantic activation can occur and remain robust at 500 ms but may dissipate before 1000 ms. The study contributes valuable evidence for the automaticity and time course of embedded semantic radical processing in Chinese phonogram recognition, thereby enhancing our understanding of sub-lexical semantic processing in logographic writing systemse.

## Introduction

**Figure 1:**
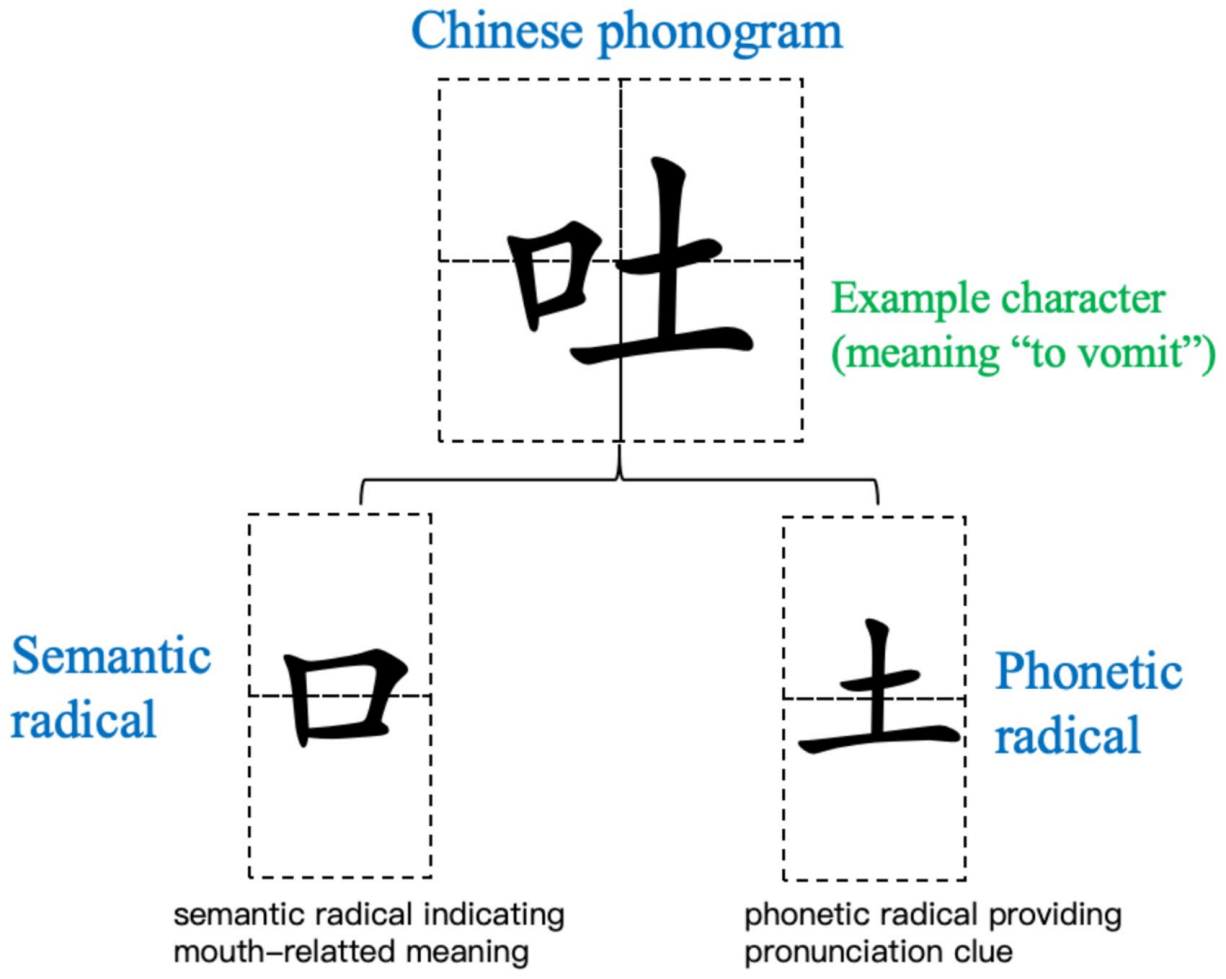
Examples of Chinese semantic-phonetic compounds and their radical components. The example character is selected based on balanced radical position (left semantic, right phonetic) and high radical frequency in modern Chinese.

Chinese adopts a unique non-alphabetic logographic writing system in which more than 80% (5,631 out of 7,000 popular words; Dictionary Compilation Office, Institute of Linguistics, Chinese Academy of Social Sciences, 2005) of Chinese characters are believed to be phonograms or phonetic compound characters. A phonogram is composed of phonetic radical (providing pronunciation cues) and semantic radical (conveying meaning) (Lee et al., 2006; Wang, 2006). For example, in “吐”(/tu3/, to spit), the embedded semantic radical “口”(/kou3/, mouth) hints at the action’s effector, while the phonetic radical “土”(/tu3/, soil) provids cues to the compound character’s pronunciation (see Figure 1 for illustration). A large number of studies have confirmed that phonetic radicals activate sub-lexical phonological (Zhou and Marslen-Wilson, 1999a, 1999b; Hsu et al., 2009; Hung et al., 2014) and semantic information (Zhou and Marslen-Wilson, 1999a; Zhou et al., 2014) during character processing, with effects emerging as early as 57–243 ms. However, whether embedded semantic radicals exhibit similar activation patterns remains unclear, given their divergent properties (e.g., combinability, frequency, positional consistency; Wang et al., 2010; Harbaugh, 1998).

### Inconsistent semantic radicals’ activation

Theoretical models provide conflicting predictions about the sub-lexical semantic activation of embedded semantic radicals (Zhou and Marslen-Wilson, 1999b; Perfetti et al., 2005; Taft, 2006; Zhang and Peng, 1993). The *Hierarchical view* indicates that embedded semantic radicals can be analyzed before the whole characters at the orthographic level, and have their own semantic information activated at an early stage of character processing. For example, Hierarchical Model posits that embedded character semantic radicals activate their meaning early via lexical-level representations of standalone radicals, while non-character radicals cannot access their meaning (Taft et al., 2000; Ding et al., 2004). Similarly, the two-network model (Zhang and Peng, 1993) proposes that phonogram recognition progresses from strokes to radicals to whole characters, with embedded semantic radicals directly accessing category meanings before host phonogram activation. Its modified versions all argue that embedded semantic radicals can have their independent form-meaning mapping in the processing of host phonograms (Chen and Zhang, 2012; Fang and Zhang, 2009). While the *paralleling view* of radicals and phonograms processing (Zhou and Marslen-Wilson, 1999a) suggest that radicals and whole characters activate orthographic, phonological, and semantic representations simultaneously. Importantly, the orthographic representations of embedded radicals can be activated and mapped onto their own corresponding phonetic and semantic representations in a parallel way as that of the whole characters. Accordingly, it can be inferred that the sub-lexical semantic activation can occur in a similar way as the lexical semantic activation process. Conversely, the *inactivation view* posits the inactivation of the sub-lexical semantic information of embedded semantic radicals. For example, lexically mediated pathways (Law et al., 2005) argue that embedded semantic radicals undergo grapheme-to-phoneme conversion without direct semantic access (Plaut et al., 1996), making radical processing primarily a phonological/orthographic event.

Both the sub-lexical activation and inactivation assumption can find support from a sizable number of empirical studies. There are several plausible empirical tests that provide evidence for the *activation* prediction. For instance, Williams and Bever (2010) found category-related semantic radicals facilitated responses (874 ms) while unrelated radicals inhibited categorization, indicating embedded semantic radicals’ automatic activation. Studies employing non-semantic oriented tasks further indicated this automatic activation. Feldman and Siok (1999a) found facilitation (43 ms SOA) when primes-targets shared radicals and semantics (R + S+), but inhibition (243 ms SOA) when sharing radicals only (R + S-), suggesting the automatically activated semantic information of embedded semantic radicals. Moreover, Zhou et al. (2013) found semantic radicals embedded in low-frequency primes facilitated target naming when sharing semantic associations, which indicated that only the semantic radicals embedded in low-frequency characters can have their semantic information activated automatically. In contrast, an array of studies also exist which observed no semantic activation of these sub-lexical units themselves (Lin et al., 2015; Wang et al., 2017; Chen and Weekes, 2004; Bi et al., 2007; Law et al., 2005). For instance, Lin et al. (2015) found real hand-radical characters activated premotor cortex more than water-radical ones, but pseudo-characters employing hand-radical and water-radical showed no difference, suggesting the failure of sub-lexical semantic activation. Similarly, Chen and Weekes (2004) found no response time differences between transparent and opaque as well as high/low-consistency characters, suggesting semantic radicals’ meaning was not activated during processing. Tong et al. (2021) demonstrated graded semantic priming effects in Chinese character recognition, showing that radical-to-character semantic relatedness (from strong to unrelated) systematically modulates radical’s activation strength across SOAs, providing compelling evidence that semantic radical activation is not only automatic and robust, but also sensitive to degrees of semantic relatedness, further enriching the interpretation of radical-based semantic access during Chinese character recognition.

### Inconsistent duration of sub-lexical semantic activation

If the semantic information of embedded semantic radicals can be activated in the character recognition, another issue arising to be addressed is how long the sub-lexical semantic activation can last, which remains theoretically underexplored. Some empirical studies, however, may shed light on this issue. For example, Flores d’Arcais et al. (1995) found that semantic radicals showed facilitation at 60 ms SOA but shifting to inhibition at 180 ms SOA, indicating that the semantic information of character semantic radicals can be activated as early as 60 ms and that the sub-lexical semantic activation may last at least 120 ms (=180–60 ms). Chen and Zhang (2008) found that high-frequency targets following radical-sharing but semantically unrelated primes (R + S-) were responded slower than the targets following radical-different and semantically unrelated primes (R-S-) at the 72 ms SOA, while neither facilitatory nor inhibitory effects of R + S- primes were observed when the SOA was lengthened to 243 ms, suggesting that the duration of the sub-lexical activation in high-frequency character processing may be as long as 171 ms (=243–72 ms). Similarly, Chen and Zhang (2012) found the semantic information of semantic radicals embedded in low-frequency characters was activated as early as 43 ms and remained robust within 243 ms, which indicated that the duration of the sub-lexical semantic activation in low-frequency character processing could be no >200 ms (=243–43 ms). In addition, Zhang and Zhang (2016) found that response latencies to targets that had different meaning but shared the same syntactic type with their embedded semantic radicals (S-G+) were slower than that to targets sharing the same semantic and syntactic information with their embedded semantic radicals (S + G+) at SOAs 57, 157, and 314 ms, which suggested that the duration of the sub-lexical activation may be at least 257 ms (=314–57 ms).

Some studies employing the ERPs techniques may provide more direct evidence concerning the duration of the sub-lexical semantic activation. For example, Wang et al. (2017) observed reduced P200 (160–230 ms) and enhanced N400 (320–420 ms) for transparent characters, suggesting easier semantic extraction (P200) followed by sustained activation (N400), and the sub-lexical semantic activation could occur within 160 ms and sustain until 420 ms, thus the duration of the sub-lexical semantic activation was at least 260 ms (=420–160 ms). Similarly, Zou et al. (2019) found that character semantic radicals elicited sequential P200 (150–250 ms), N400 (300–400 ms), and LPC (550–650 ms), indicating the sub-lexical semantic activation may occur within 300 ms and endure until 650 ms, with the duration about 350 ms (=650–300 ms).

Taken together, while evidence increasingly supports semantic radical activation, the duration of semantic activation of embedded semantic radicals has not been investigated in adequate detail. Based on what is reviewed in the foregoing, some basic summaries can be made: (1) embedded semantic radicals are more likely to have their own semantic information activated than not, but there is no firm conclusion; (2) once the sub-lexical semantic information is activated, it can last for a time, with the most possible duration ranging from 120 ms to 350 ms (e.g., Flores d’Arcais et al., 1995; Zhang and Zhang, 2016; Chen and Zhang, 2008, 2012; Zou et al., 2019); (3) beyond the possible time window of sub-lexical semantic activation event enclosed by an onset time and an offset time, the activation is difficult to capture. On the basis of previous studies, a tentative conjecture can be made that the sub-lexical activation event may set on at a time no later than 100 ms, such as 43 ms (Feldman and Siok, 1999a), 57 ms (Zhang and Zhang, 2016), 60 ms (Flores d’Arcais et al., 1995), 72 ms (Chen and Zhang, 2008). And the sub-lexical activation may set off at a time no later than 1,000 ms, such as 650 ms (Zou et al., 2019) and 874 ms (Williams and Bever, 2010). Accordingly, the sub-lexical activation may start as early as 43 ms, and may vanish no later than 1,000 ms, which indicated that the interval of 43–1,000 ms could be the rough time window in which the semantic activation of embedded semantic radicals is likely to be captured.

However, three key gaps persist. First, the 43–1,000 ms window remains underexplored, with fragmentary data at 180/243/314 ms SOAs. Second, existing duration estimates vary widely (120 vs. 350 ms) due to methodological differences in tasks and materials. Third, the mechanisms driving late-stage effects (e.g., LPC at 550–650 ms; Zou et al., 2019) lack systematic investigation. Crucially, no study has concurrently examined the full activation spectrum from 100 to 1000 ms using comparable paradigms. Since the time interval of 43–100 ms has been relatively thoroughly explored with SOAs 43, 57, 60, 72, and 100 ms, the current study addresses these gaps by testing the less explored time interval of 100–1,000 ms. Given that a few studies, as mentioned previously, have investigated the issue at SOAs 180, 243, and 314 ms, it is tempting to further examine the issue by experimenting with another three SOAs, namely 100, 500, and 1,000 ms. SOA 100 ms is expected be an approximate time-point to gauge the minimum duration of the activation. If the earliest sub-lexical semantic activation of radicals can occur before 100 ms, no matter at 43 ms, 57 ms, 60 ms or 72 ms, the starting time plus the minimal duration is likely to exceed 100 ms. Therefore, 100 ms SOA may serve as the smallest time window in which the sub-lexical semantic activation may be observed. Similarly, SOA 500 ms may function as an approximate time-point to gauge the typical duration of a sub-lexical semantic activation event. Since previous studies suggested that the sub-lexical semantic activation may occur before 100 ms and can last at least 120–350 ms (e.g., Flores d’Arcais et al., 1995; Zhang and Zhang, 2016; Zou et al., 2019), the possible time window with the typical duration may be 220–450 ms within which the sub-lexical activation can be observed. Wang et al. (2017) and Zou et al. (2019) also lend support to this assumption because they found that the semantic information of embedded semantic radicals remained active around 400 ms (as indicated by the N400) and even around 600 ms (as indicated by the LPC). As for SOA 1000 ms, it may function as an approximate time-point to gauge the maximum duration of the semantic activation of embedded semantic radicals. Previous studies showed that the sub-lexical semantic activation may end before 1,000 ms (e.g., 874 ms, Williams and Bever, 2010) to ensure the accurate recognition of the whole characters, since single character processing is likely to be finished before 1,000 ms (Perfetti and Tan, 1998; Lin and Han, 1999). For example, when participants were asked to judge whether the targets corresponded with the previously presented description concerning the orthographic, phonetic and semantic features of the targets, Lin and Han (1999) found that mean reaction times to semantic coding condition were 718 ms, which indicated that the semantic analysis of characters can be completed within 718 ms.

### The present study

The present study, by employing a 2 (prime-target relatedness: related vs. unrelated) × 3 (SOA: 100 ms vs. 500 ms vs. 1,000 ms) × 2 (prime frequency: high vs. low) between-subjects design, aimed to address two questions: (1) Whether embedded semantic radicals can automatically activate their semantic meaning during phonogram recognition? (2) What is the precise temporal dynamics of such activation? Using a priming character decision task in which participants were asked to decide whether a target item was a legal character or not, response accuracy and reaction time differences were measured. The targets were only semantically related to the semantic radicals embedded in the related primes but not the primes themselves. Therefore, any priming effect in related prime condition can directly demonstrate the semantic activation of embedded semantic radicals. Importantly, we varied the interval between prime and target (SOA) to gauge the duration of the sub-lexical activation: 100, 500, and 1,000 ms. These SOAs are intended to capture different stages of cognitive processing during masked priming. A 100 ms SOA reflects rapid, automatic pre-lexical activation before conscious awareness (e.g., Grainger et al., 2016). A 500 ms SOA allows sufficient time for lexical-semantic integration and conscious recognition processes to take place (e.g., Estes and Jones, 2009). Meanwhile, the 1,000 ms SOA likely reflects later-stage processing, including semantic inhibition or suppression effects as the activation decays or is re-evaluated (e.g., Piai et al., 2014).

It was expected that the facilitatory effect of related primes which indicated sub-lexical semantic activation can be observed within the minimum time window (100-ms SOA) and the typical time window (500-ms SOA), but not necessarily at the maximum time window (1000-ms SOA) since the sub-lexical activation may have been completed within this time interval. In addition, the frequency of primes were manipulated in order to further explore whether the occurrence and duration of the sub-lexical activation was sensitive to the frequency of their host phonograms as indicated by some studies (e.g., Zhou et al., 2013; Chen and Zhang, 2008, 2012).

## Methods

### Subjects

An *a priori* power analysis was conducted using G*Power 3.1 (Faul et al., 2007) to determine the required sample size for a mixed-design ANOVA, which included one between-subjects factor (SOA: 100, 500, 1,000 ms) and two within-subjects factors (prime type: related vs. control; radical frequency: high vs. low). To detect a medium effect size (*f* = 0.25) with 80% power at an alpha level of 0.05, the analysis indicated that a total of 30 participants (10 per SOA group) were needed. To account for potential exclusions due to low data quality, we recruited a total of 117 participants (all females, aged 22–30 years, mean age = 25.05 years, SD = 1.91). The participants were randomly divided into three groups, 39 for the 500 ms SOA condition, 39 for the 100 ms SOA condition and another 39 for the condition of SOA 1000 ms. All participants were undergraduate or graduate students recruited from Sichuan International Studies University (SISU). They were all native Mandarin speakers who grew up in Mainland China, and all were right-handed, with the normal or corrected-to-normal vision. Before participation, participants were asked to provide written informed consents and got paid after the experiments.

### Materials and design

Stimuli used in this study were similar to those used in the Experiment 1 carried out by Zhou et al. (2013). However, three significant revisions were made to Zhou et al. (2013) experimental materials. Firstly, the radical “月” used by Zhou et al. (2013) were excluded, since this semantic radical indicates “flesh-related” meaning while the simple character “月” indicates the “moon” meaning. The consideration is that differences in the semantic information indicated by the same graphic form at lexical and sub-lexical level may spoil the semantic relation between targets and semantic radicals embedded in the primes, thereby confusing the interpretation of experimental results. Secondly, some control characters (e.g., “犄”) whose embedded semantic radicals may share semantic relatedness with the corresponding targets (e.g., “耕”) were also replaced to ensure unambiguous semantic relationships. This replacement followed three criteria: (1) semantic transparency (the radical’s meaning consistently contributes to the host phonogram’s meaning), (2) radical productivity (selecting radicals that appear in multiple characters with stable semantic contributions), and (3) visual discriminability (avoiding radicals with highly similar visual forms that might cause orthographic confusion). Thirdly, some related primes with the embedded semantic radicals on the right-side were also rejected, since radicals on the right-side are less likely to be treated as semantic radicals (Wu et al., 2013; Ding et al., 2004). This left-side bias selection was further justified by that the statistical predominance of left-positioned semantic radicals in Chinese phonograms (approximately 90% of semantic radicals appear on the left; Hsiao and Shillcock, 2006).

Therefore, in the present study, a total of 48 pairs of phonograms with embedded character semantic radicals on the left served as primes, of which 24 pairs served as high-frequency primes (Mean = 192.52 per million, SD = 190.70 per million) and 24 pairs served as low-frequency primes (Mean = 4.16 per million, SD = 3.99 per million) based on the corpus of the Centre for Chinese Linguistics at Peking University (CCL). This classification followed the established method of using the upper and lower quartiles of radical frequency distributions obtained from the CCL corpus (Zhou et al., 2013; Zhang and Zhang, 2016). In the high-frequency condition, the related primes and control primes had no significance in terms of frequency (*t* = 1.55, *p* = 0.136) and number of strokes (*t* = −0.55, *p* = 0.59). Similarly, in the low-frequency condition, these two kinds of primes did not differ from each other in terms of frequency (*t* = 0.945, *p* = 0.354) and strokes (*p* = 0.11; *p* = 0.30). Related primes and their corresponding control primes shared the same phonetic radical and regularity.

To make sure that the expected priming effects can only be attributed to the semantic radicals embedded in related primes rather than the related primes themselves, the semantic transparency of both related primes and control primes were rated by 30 participants (all females) who did not take part in the formal experiments on a 5-point scale questionnaire ranging from 1 (extremely opaque) to 5 (extremely transparent). In the high-frequency condition, the average scores for related primes and control primes were 1.97 and 2.45, respectively. In the low-frequency condition, the average scores for the related primes and control primes were 1.96 and 2.5, respectively. The relative low rating scores of related primes (below 2 points) indicated that they may be characterized into semantic opaque characters.

Forty-eight targets (24 preceded by high-frequency primes and 24 by low-frequency primes) were then selected. These targets were only semantically related with the semantic radicals embedded in related primes, but not with the related primes themselves. For example, the target character “男”(/nan2/, male) shared no semantical similarity with the high-frequency related prime “嫌”(/xian2/, dislike), but it was semantically related with the semantic radical “女” (/nv3/, female) embedded in “嫌” (see Table 1 for sample stimuli). No orthographic or phonetic similarities exist between targets and primes. Moreover, the targets had nothing in common with the control primes (e.g., “赚”/zhuan4/, profit). The targets in high-frequency-prime condition and that in low-frequency-prime condition had no significant differences in terms of frequency (*t* = −1.41, *p* = 0.173) and strokes (*t* = 0.54, *p* = 0.596).

**Table 1:** Sample stimuli of primes and targets.

Frequency	Type of component	Priming condition		Target
		Related	Control	
High-frequency	Phonogram	嫌 (xian2:dislike)	赚 (zhuan4: profit)	男 (nan2: male)
	Semantic radical	女 (nv3: female)	贝 (bei4: shellfish)	
	Phonetic radical	兼 (jian1: double)		
Low-frequency	Phonogram	赅 (gai1: full)	骇 (hai4: astonished)	蚌 (bang4: mussel)
	Semantic radical	贝 (bei4: shellfish)	马 (ma3: horse)	
	Phonetic radical	亥 (hai4: the last of the 12 Earthly Branches)		

In order to make sure that the targets only shared semantic association with the semantic radicals embedded in the related primes, the same 30 participants also rated the semantic association between targets and two kinds of primes as well as the semantic relatedness between targets and semantic radicals embedded in the two kinds of primes on a 7-point scale questionnaire. In the high-frequency condition, the semantic relatedness between targets and related primes and that between targets and control primes had no significant difference (*t* = −0.037, *p* = 0.971). Similarly, in the low-frequency condition, the semantic relatedness between targets and the two kinds of primes had no significant difference (*t* = 0.199, *p* = 0.844). Besides, the semantic relatedness between targets and semantic radicals embedded in related primes and that between targets and semantic radicals embedded in control primes had significant difference both in the high-frequency condition (*t* = 80.01, *p* = 0.00) and in the low-frequency condition (*t* = 112.51, *p* = 0.00). Furthermore, we ensured that no phonological similarity existed between the primes and targets by excluding any pairs that shared syllables, onsets, rhymes, or tones. This was confirmed by three independent native Chinese speakers.

In addition, 48 pairs of pseudo-characters were created as fillers by combining semantic and phonetic radicals in position-illegal manner. Therefore, a total of 192 prime-target pairs were used for high-frequency and low-frequency conditions (see Appendix Tables 2, 3 for all materials).

### Procedure

**Figure 2:**
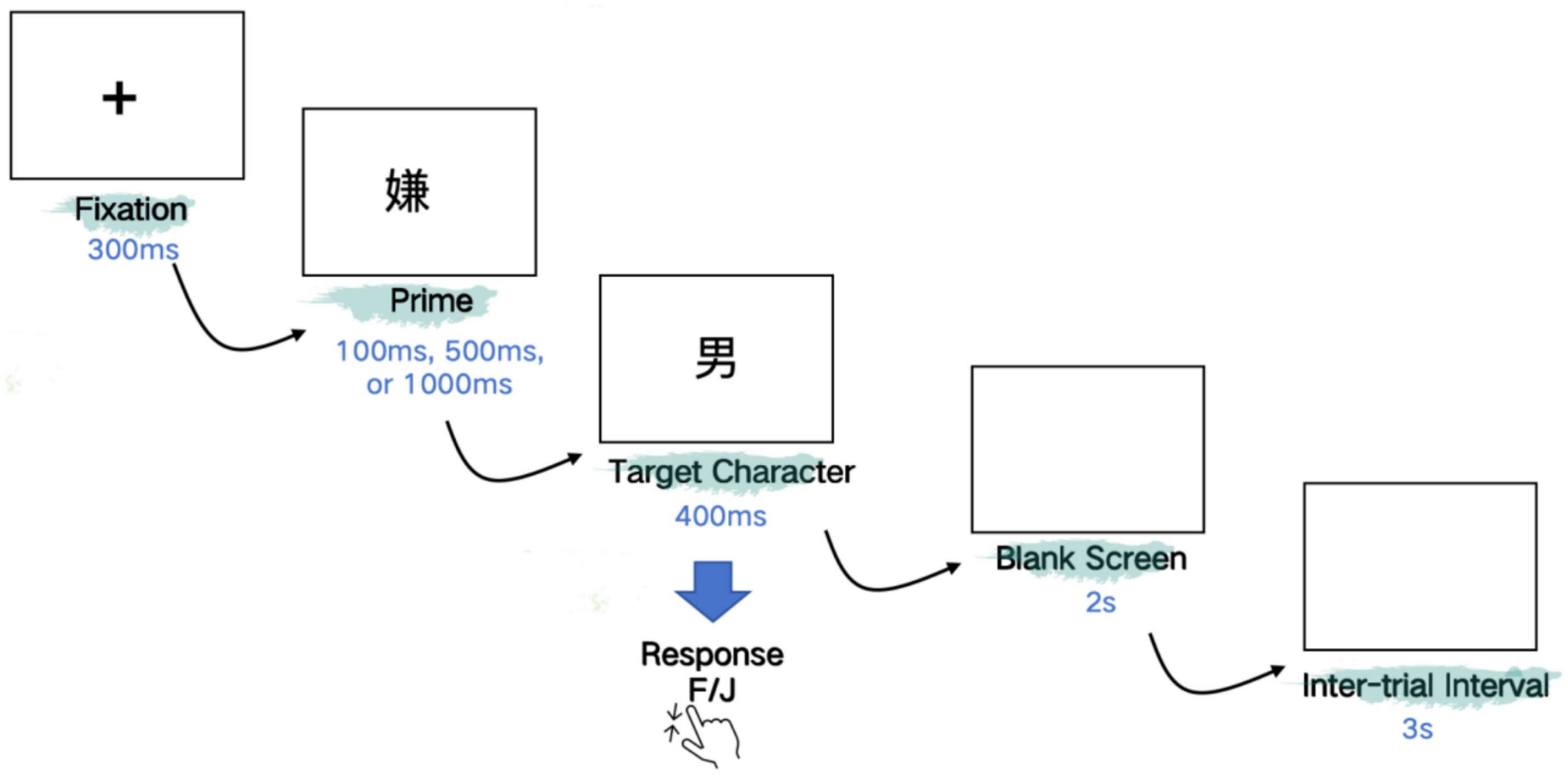
A detailed overview of the experimental trial structure with timing parameters.

Each trial began with a fixation “+” displayed in the center of the screen for 300 ms in order to draw the participants’ attention. Then a prime was presented for 100 ms, 500 ms or 1,000 ms respectively, depending on the SOA condition. Primes were replaced immediately by a target with the duration of 400 ms. The target was followed by a blank that remained for 2 s and the inter-trial interval was 3 s (see Figure 2 for illustration). Participants were asked to judge whether the presented item was a real Chinese character or not as quickly and accurately as they can after the onset of targets, with the accuracy and reaction time being recorded. For half of the participants, the key “F” indicated a “YES” response and “J” a “NO” response. The keys of response were reversed for the other half of the participants. Both primes and targets were displayed in font Song and in black against a white background. Before the presentation of the experimental materials, 10 additional pairs of primes and targets were presented for practice. And during the experiments, participants could have a rest every 48 trials. All experiments were controlled by the software E-Prime 2.0 (Psychology Software Tools, Inc.) and conducted in the Key Laboratory of Cognitive Neuroscience and Foreign Language Learning in SISU.

## Results

Data of one subject for the 100 ms SOA condition and data of two subjects for the 500 ms SOA condition were rejected from the final analysis for the quitting of participating. Reaction times of incorrect responses and the extreme data beyond 2.5SD from the mean RT of each condition were excluded from data analysis, with >5% of total trials being rejected.

A three-way mixed design repeated-measures ANOVA was employed to measure the RTs by subjects (*F*_1_) and by items (*F*_2_). In the by-subject analysis, the RELATENDESS (related vs. control primes) and FREQUENCY (low vs. high-frequency primes) were treated as within-subject factors, and the SOAs (100 ms vs.500 ms vs. 1,000 ms) was treated as the between-subject factor. In the by-item analysis, the relatedness and SOAs were treated as within-item variables, and the frequency was treated as the between-item variable. The mean reaction times to target in each prime condition were summarized in Table 2.

**Table 2:** Mean response latencies (ms) for targets.

Frequency	100 ms SOA		500 ms S0A		1,000 ms SOA	
	Related primes	Control primes	Related primes	Control primes	Related primes	Control primes
High-frequency	560.47 ± 79.28	559.57 ± 80.12	597.66 ± 104.69	598.34 ± 116.12	604.79 ± 102.87	607.17 ± 109.53
Low-frequency	555.34 ± 81.38	561.59 ± 78.41	583.07 ± 106.37	596.73 ± 115.37	598.64 ± 105.41	592.67 ± 103.77

There was a significant main effect of FREQUENCY in the by-subject analysis, *F_1_* (1, 111) = 9.836, *p* = 0.002, MSE = 513.85, *η*^2^ = 0.081, but no significant effect was observed in the by-item analysis, *F_2_* (1, 46) = 1.375, *p* = 0.247, MSE = 354.59, *η*^2^ = 0.029. The main effect of RELATEDNESS was not significant either in the by-subject analysis, *F_1_* (1, 111) = 1.162, *p* = 0.283, MSE = 706.72, *η*^2^ = 0.010, or in the by-item analysis, *F_2_* (1, 46) = 2.509, *p* = 0.120, MSE = 372.233, *η*^2^ = 0.052. Besides, the main effect of SOA was insignificant by subject, *F_1_* (2, 111) = 1.999, *p* = 0.140, MSE = 9485.36, *η*^2^ = 0.035, but was significant by item, *F_2_* (2, 92) = 108.079, *p* = 0.000, MSE = 538.831, *η*^2^ = 0.701. The interaction effect between RELATEDNESS, FREQUENCY and SOA was marginally significant both by subject (*p_1_* = 0.087) and by item (*p_2_* = 0.082). No other significant interaction was observed either by subject or by item.

**Figure 3:**
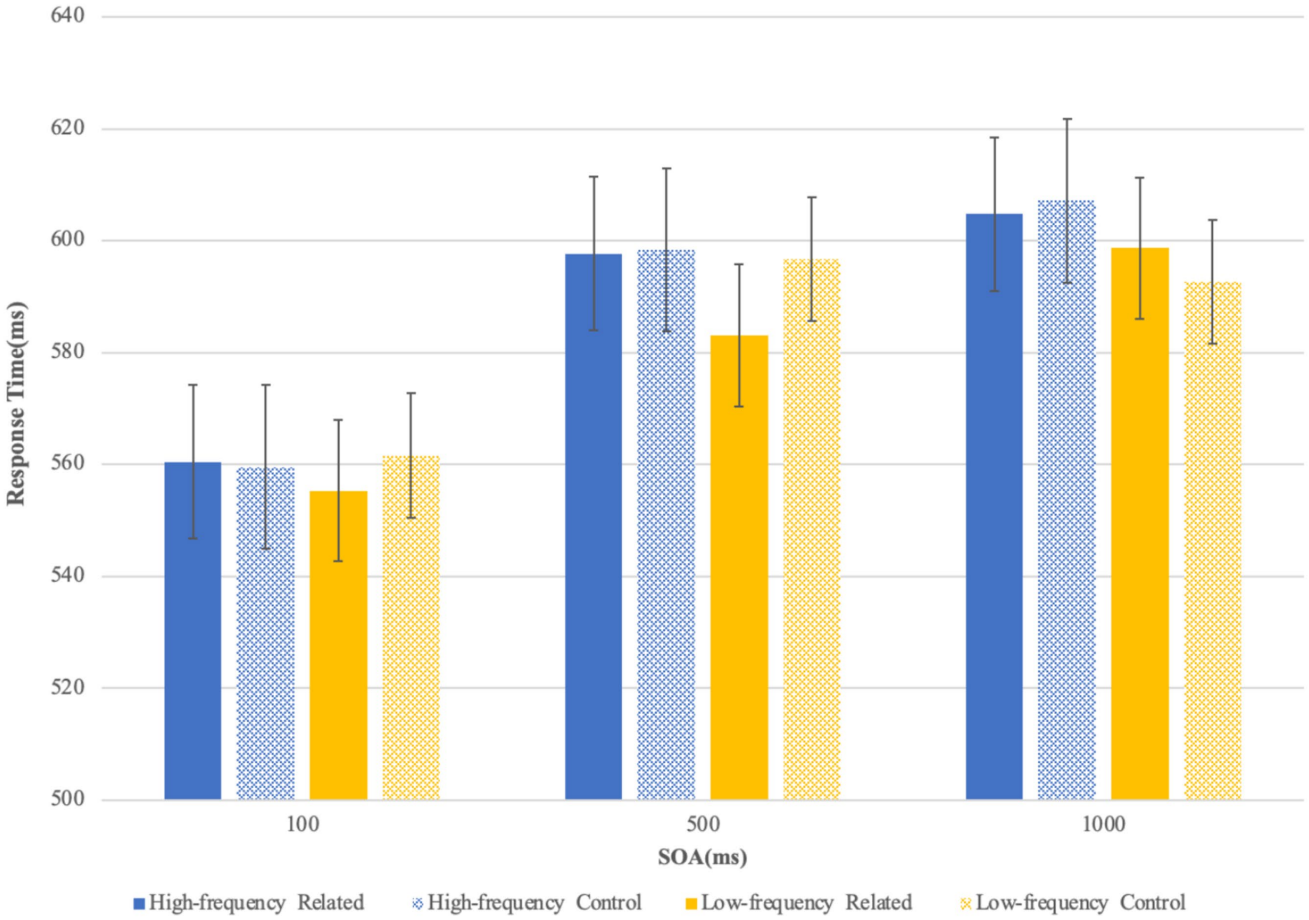
Results of the semantic primed character decision experiment (the *bars* represent standard deviation).

The simple effect analysis showed that in the 100-ms SOA condition, the RTs to targets preceded by related primes did not significantly differ from that preceded by control primes, either in the high-frequency condition [*F_1_* (1, 111) = 0.02, *p* = 0.977; *F_2_* (1, 46) = 1.16, *p* = 0.288] or in the low-frequency condition [*F_1_* (1, 111) = 1.39, *p* = 0.241; *F_2_* (1, 46) = 0.74, *p* = 0.393]. In the 500-ms SOA, RTs to targets following high-frequency related primes did not differ from that following high-frequency control primes [*F_1_* (1, 111) = 0.01, *p* = 0.907; *F_2_* (1, 46) = 0.04, *p* = 0.834]. However, the low-frequency related primes, compared with low-frequency control primes, significantly facilitated the target decision in the 500-ms SOA [*F_1_* (1, 111) = 6.44, *p* = 0.013; *F_2_* (1, 46) = 6.23, *p* = 0.016; Cohen’s d = 0.44; *η*^2^ = 0.055 (by subject), *η*^2^ = 0.119 (by item)] (see Figure 3). Moreover, in the 1,000-ms SOA, RTs to targets preceded by related primes did not significantly differ form that following control primes either in the high-frequency condition [*F_1_* (1, 111) = 0.17, *p* = 0.678; *F_2_* (1, 46) = 2.05, *p* = 0.159] or in the low-frequency condition [*F_1_* (1, 111) = 1.30, *p* = 0.257; *F_2_* (1, 46) = 0.14, *p* = 0.713].

## Discussion

Previous studies have noted the importance of embedded semantic radicals in phonogram recognition. This study utilized the SP phonograms to examine the issue of the duration of the embedded semantic radicals through three selected SOAs (100, 500, and 1,000 ms).

At SOA 100 ms, neither the high-frequency related primes nor the low-frequency related ones facilitated the response to targets, which indicated that the semantic radicals embedded in related primes had not activated their own semantic information. This absence of the sub-lexical semantic activation runs direct counter to Zhou et al. (2013) who reported facilitatory priming effect on targets following low-frequency related primes at SOA 100 ms. The main difference between the two studies is that while they used lexical naming task, we adopted character decision task. Although it seems that these two tasks are both semantically irrelevant, in deciding whether the target was a true Chinese character or not, the subjects’ attention was primarily focused on the orthographic analysis, such that the embedded semantic radicals were more likely to be involved in mediating the character representations, with their own sub-lexical semantic information less likely to be activated or the activation being rendered too weak to be detectable (Zou et al., 2019). The lack of facilitation for high-frequency primes may also be due to their frequent appearance in semantically opaque compounds, leading to diminished activation of the radical’s sub-lexical semantics. Frequent radicals may become automatized in lexical processing, with their form being recognized without reactivating the underlying semantic feature, a phenomenon akin to semantic satiation (Taft and Zhu, 1997). Moreover, high-frequency radicals often appear in opaque characters, where their semantic contribution is weakened or overridden by lexical whole-word processing (Feldman and Siok, 1999b). This explanation aligns with our transparency ratings, where related primes (both high and low frequency) scored below 2, suggesting low transparency. It is also possible that the difference is ascribable to the three revisions made to the experimental materials, as mentioned previously.

This result might indicate that the embedded semantic radicals did not activate their semantic information, as suggested by the *inactivation view* (Law et al., 2005) and supported by a host of previously-mentioned empirical studies showing the inactivation of sub-lexical units (e.g., Lin et al., 2015; Wang et al., 2017; Chen and Weekes, 2004). This account is further strengthen by the reporting that semantic analysis was found to occur later than orthographic and phonological analyses in character processing (Xu and Deng, 2011; Perfetti and Tan, 1998; Tan et al., 1995). For example, in a priming naming experiment, Perfetti and Tan (1998) found the facilitatory effects of graphic primes at SOA 43 ms and the facilitation of homophonic primes at 57 ms SOA, while the semantic primes showed significant facilitation at 115 ms SOA. Similarly, with a backward-masking procedure, Tan et al. (1995) found that when character targets were presented for 50 ms, followed by the masks presented for 30 ms that shared orthographic, phonological or semantic similarities with the targets, only graphic but not phonological nor semantic masks affected target recognition. While when the exposure durations of the target and mask were extended to 60 and 40 ms respectively, a significant phonological mask facilitation effect on high-frequency targets was observed but the semantic mask effect was still absent. The result is also especially strengthened when we consider a number of studies that have reported much later sub-lexical semantic activations, for example, SOA 243 ms (Chen and Zhang, 2008, 2012; Feldman and Siok, 1999a) and SOA 314 ms (Zhang and Zhang, 2016).

From another perspective, the sub-lexical semantic activation of embedded semantic radicals, and/or the duration of the activation may depend on a myriad of factors which function vitally in this process, ranging from the properties (e.g., frequency, combinability, position, consistency, syntactic information) of radicals themselves, to those (e.g., frequency, transparency, consistency, structure) of the embedding host phonograms under processing. This may help explain why the sub-lexical semantic activation effects have been found at such diverse time-points as manifested in the range of SOAs. Given the design of the present study, however, what can be said is that the utility of the time-point SOA 100 ms remains uncertain in gauging the minimal size of the duration of the radical’s sub-lexical semantic activation event.

At SOA 500 ms, the response latencies to targets preceded by low-frequency related primes were faster than those to targets following control primes. This facilitatory effect demonstrated that the free-standing semantic radicals can access their own semantic information prior to 500 ms and this sub-lexical activation can remain robust around 500 ms. This finding goes in accordance with the few ERPs studies which have reported the sub-lexical radical semantic activation around 500 ms as indicated by the N400 and LPC (Wang et al., 2017; Zou et al., 2019). As such, SOA 500 ms supposedly serves as an approximate time-point to gauge the typical size of a sub-lexical semantic activation duration in phonogram processing, especially when the semantic radicals were embedded in low-frequency characters.

In contrast to low-frequency condition, no facilitatory effect was observed for high-frequency related primes at SOA 500 ms. This frequency-biased sub-lexical semantic activation was in line with Zhou et al. (2013) and Chen and Zhang (2012) which both reported modulating effect of lexical frequency in semantic processing of radicals. It was assumed that the semantic activation of radicals embedded in high-frequency characters may be relatively weak due to the different strategies employed in high- and low-frequency character recognition as suggested in the models (e.g., two-network model) following the hierarchal view (Zhang and Peng, 1993; Chen and Zhang, 2012). To be specific, the high-frequency words are read “logographically,” while low-frequency words “analytically” (Seidenberg, 1985). Analytical reading means that the embedded semantic radicals are more likely to be extracted from the host phonograms and linked to their own semantic representations. Logographical reading, in contrast, means that they are more involved in mediating the lexical representations of the whole characters rather than mapping to their own meanings, such that the sub-lexical semantic activation may be rendered too weak to be detected.

With regard to SOA 1000 ms, no facilitatory effect was observed for either high-frequency or low-frequency primes, which may indicate the occurrence of a suppression of the sub-lexical semantic activation of radicals. According to theoretical models which uphold hierarchical view, like the hierarchical model (Taft, 2006), though the character embedded semantic radicals can activate their corresponding semantic representations, the final semantic outputs are oriented toward the whole character, not toward the constituting components. Once the sub-lexical semantic information is integrated into the meaning of the whole character, the sub-lexical activation may not be maintained any more. As for the present study, the related primes were semantically opaque phonograms where the semantic information of embedded semantic radicals had no relatedness with the meaning of the whole character, thus the sub-lexical semantic activation would be expected to be suppressed even readily prior to the execution of the semantic analysis of the host phonograms. An alternative account for this null effect at 1000 ms considers the role of attention. Studies using rapid serial visual presentation (RSVP) have shown that semantic priming diminishes when attention is diverted (e.g., Luck et al., 1996). Thus, prolonged intervals may allow attention to shift away from prime processing entirely, rendering any initial radical activation irrelevant to target judgment.

In the literature, no research was found to have specially addressed the issue of whether or not the radical’s sub-lexical activation could be suppressed at SOA 1000 ms. A likely study is Lin et al. (2015) who found that when the pseudo-characters with hand radical “扌” was presented for 1,000 ms, the premotor cortex was still inactivated. This result, of course, indicated that the embedded semantic radical failed to activate its action-related meaning. But an equally possible interpretation was that within 1,000 ms, the action-related meaning had been activated and then suppressed. Support for this speculation seems to come from Williams and Bever (2010) who demonstrated that the radical’s semantic activation occurred within 874 ms, as mentioned in the foregoing. It is assumed that the sub-lexical activation set on at a time earlier than 874 ms, and may be suppressed around 874 ms, otherwise the semantic categorization of the whole characters cannot be accurately completed. The absence of facilitatory effects at SOA 1000 ms in our study, therefore, might suggest that SOA 1000 ms exceeds the maximum size of the duration of the radical’s semantic activation.

The processing role of radicals in character recognition has always been of focal interest to researchers. A couple of theoretical models (e.g., Taft, 2006; Zhang and Peng, 1993; Zhou and Marslen-Wilson, 1999a) have predicted sub-lexical contributions of embedded semantic radicals to the lexical-level semantic access of host characters, presuming the occurrence of their semantic activation. From the viewpoint of Chinese acquisition, it should be a matter of nature that Chinese readers analyze the meaning of embedded semantic radicals when recognizing a phonogram since embedded semantic radicals are always extracted from host phonograms for detailed analysis and the meanings of the embedded semantic radicals are directly taught to learners (Nguyen et al., 2017). In the meantime, numerous studies have investigated the sub-lexical activation issue, but very few studies have focused on how the sub-lexical activation event occurs and proceeds. The size of the time window of embedded semantic radicals’ semantic activation remains largely unexplored. It is intriguing to characterize the minimum, the typical, and the maximum duration of such an activation event, which will presumably provide new insights into Chinese character processing. The present study, by employing three SOAs, namely, 100 ms, 500 ms and 1,000 ms, made an effort in this respect. The results seem to suggest that while the status of SOA 100 ms and SOA 1000 ms remain uncertain in gauging, respectively, the minimum and maximum duration of the radical’s semantic activation event in phonogram recognition, SOA 500 ms may serve as an approximate time-point to gauge the typical duration of the sub-lexical activation event.

Though the results and findings of the present study are meaningful and worthwhile, future research efforts are needed. Not only are replication studies of the present study needed in the future, studies that employ more fine-grained SOAs lying between 100–500 ms and 500–1,000 ms (e.g., 750 ms) are especially expected, in order to provide more detailed information concerning the occurrence and the duration of the sub-lexical semantic activation. It is also recommendable that future researches take into account the properties of embedded semantic radicals, such as consistency and frequency, which may bear on the processing of host phonograms (Chen and Weekes, 2004). Moreover, a shorter inter-trial interval, a more gender-balanced sample and some complementary paradigms (e.g., semantic categorization task or lexical naming task) could be considered in future research to rule out potential confounding effects. Future studies could also further explore the role of orthographic structure in semantic activation by systematically manipulating the position of the semantic radical (e.g., left vs. right) within the character. Additionally, examining the effects of semantic concreteness—both of radicals and of entire characters—may help uncover how abstractness modulates the depth and speed of semantic processing. These directions could provide new insights into the interface between visual-orthographic features and semantic representation in Chinese character recognition.

## Conclusion

The present study, by employing character decision task, probed into the automatic semantic activation of embedded semantic radicals and the duration of the sub-lexical activation. For this purpose, the semantic relatedness between targets and semantic radicals embedded in primes, frequency of host phonograms as well as SOAs for primes were manipulated. It was found that the semantic information of embedded semantic radicals was activated at SOA 500 ms, but only for low-frequency host phonograms, exhibiting a modulating effect of lexical frequency. At the SOA of 100 and 1,000 ms, sub-lexical semantic activation was found for neither. These results indicate that SOA 500 ms functions as an appropriate time-point to gauge the typical size of the duration of the sub-lexical activation, while SOA 100 ms and SOA 1000 ms are still uncertain as time-points to, respectively, gauge the minimum and maximum size of the duration of the embedded semantic radical’s sub-lexical activation. However, the present study takes just a preliminary effort in revealing how the radical’s sub-lexical semantic activation sets on and proceeds. More elaborate studies are called for in the future.

## Data Availability

The raw data supporting the conclusions of this article will be made available by the authors, without undue reservation.
