# Supplementary material for: Will the embedded semantic radicals be activated when recognizing Chinese phonograms?
Source: Front Hum Neurosci. 2025 Jun 13;19:1550536. doi: 10.3389/fnhum.2025.1550536 (PMC12202340; doi:10.3389/fnhum.2025.1550536)
Supplement: Supplementary file 1 [file Table_1.docx]

**Appendix**

**Table 1** Stimuli of high-frequency in character decision task

| Item no | Prime type | | Semantic radical | Target |
| --- | --- | --- | --- | --- |
|  | Related | Control |  |  |
| 1 | 嫌 | 赚 | 女 | 男 |
| 2 | 始 | 抬 | 女 | 郎 |
| 3 | 坏 | 杯 | 土 | 沙 |
| 4 | 稳 | 隐 | 禾 | 米 |
| 5 | 杭 | 航 | 木 | 丛 |
| 6 | 般 | 股 | 舟 | 载 |
| 7 | 静 | 净 | 青 | 蓝 |
| 8 | 默 | 状 | 黑 | 绿 |
| 9 | 验 | 检 | 马 | 鹿 |
| 10 | 骗 | 偏 | 马 | 虎 |
| 11 | 弥 | 称 | 弓 | 箭 |
| 12 | 略 | 路 | 田 | 稻 |
| 13 | 极 | 级 | 木 | 筏 |
| 14 | 权 | 仅 | 木 | 炭 |
| 15 | 玛 | 码 | 王 | 臣 |
| 16 | 稿 | 搞 | 禾 | 田 |
| 17 | 辅 | 铺 | 车 | 舟 |
| 18 | 殊 | 珠 | 歹 | 毒 |
| 19 | 增 | 赠 | 土 | 灰 |
| 20 | 听 | 析 | 口 | 舌 |
| 21 | 校 | 较 | 木 | 炭 |
| 22 | 职 | 织 | 耳 | 鼻 |
| 23 | 较 | 校 | 车 | 骑 |
| 24 | 粗 | 组 | 米 | 食 |

**Table 2** Stimuli of low-frequency in character decision task

| Item no | Prime type | | Semantic radical | Target |
| --- | --- | --- | --- | --- |
|  | Related | Control |  |  |
| 1 | 畸 | 绮 | 田 | 耕 |
| 2 | 耽 | 枕 | 耳 | 听 |
| 3 | 娓 | 梶 | 女 | 母 |
| 4 | 赅 | 骇 | 贝 | 蚌 |
| 5 | 秤 | 抨 | 禾 | 苗 |
| 6 | 稚 | 帷 | 禾 | 谷 |
| 7 | 毓 | 梳 | 每 | 各 |
| 8 | 韶 | 貂 | 音 | 响 |
| 9 | 黩 | 椟 | 黑 | 暗 |
| 10 | 贻 | 殆 | 贝 | 壳 |
| 11 | 赅 | 垓 | 贝 | 海 |
| 12 | 轶 | 佚 | 车 | 船 |
| 13 | 墉 | 慵 | 土 | 泥 |
| 14 | 娓 | 艉 | 女 | 子 |
| 15 | 皖 | 烷 | 白 | 纯 |
| 16 | 贬 | 砭 | 贝 | 螺 |
| 17 | 瑾 | 槿 | 王 | 候 |
| 18 | 琉 | 硫 | 王 | 君 |
| 19 | 珈 | 枷 | 王 | 冠 |
| 20 | 粘 | 沾 | 米 | 谷 |
| 21 | 矜 | 衿 | 矛 | 盾 |
| 22 | 黜 | 绌 | 黑 | 乌 |
| 23 | 砍 | 炊 | 石 | 崖 |
| 24 | 骇 | 骸 | 马 | 牛 |
